# Highly Efficient Heavy Atom Free Room Temperature Phosphorescence by Host-Guest Doping

**DOI:** 10.3389/fchem.2021.781294

**Published:** 2021-11-23

**Authors:** Jinzhu Cao, Meng Zhang, Manjeet Singh, Zhongfu An, Lingfei Ji, Huifang Shi, Yijian Jiang

**Affiliations:** ^1^ Institute of Laser Engineering, Beijing University of Technology (BJUT), Beijing, China; ^2^ China Electronics Standardization Institute, Beijing, China; ^3^ Key Laboratory of Flexible Electronics (KLOFE), Institute of Advanced Materials (IAM), Nanjing Tech University (NanjingTech), Nanjing, China

**Keywords:** host-guest doping, heavy atom free, room temperature phosphorescence (RTP), triplet state, nonradiative transition

## Abstract

Recently, there has been remarkable progress of the host-guest doped pure organic room-temperature phosphorescence (RTP) materials. However, it remains a great challenge to develop highly efficient host-guest doping systems. In this study, we have successfully developed a heavy atom free pure organic molecular doped system (benzophenone-thianthrene, respectively) with efficient RTP through a simple host-guest doping strategy. Furthermore, by optimizing the doping ratios, the host-guest material with a molar ratio of 100:1 presented an efficient RTP emission with 46% quantum efficiency and a long lifetime of up to 9.17 ms under ambient conditions. This work will provide an effective way to design new organic doping systems with RTP.

## 1 Introduction

Recently, purely organic room temperature phosphorescence (RTP) materials are gaining more and more attention because of their long luminescent lifetimes, larger Stokes shift, convenient syntheses, low cost, and so on (Ma et al., 2019; Xiong et al., 2019), showing potential applications in display (Wang et al., 2019), data storage (Xu et al., 2016), encryption (Hirata, 2017), and bioimaging (Qu et al., 2019), and so on. Due to the inherent spin-forbidden and the fast-non-radiative transition of triplet excitons, it is quite hard to obtain RTP based on metal-free organic compounds. So far, in order to increase intersystem crossing (ISC) rate and to suppress non-radiative transitions, many strategies have succeeded to achieve efficient organic RTP materials, such as host-guest doping, heavy-atom effects, intermolecular electronic coupling, supramolecular self-assembly, and so forth (Yang et al., 2016; Chai et al., 2017; Cai et al., 2018; Yang et al., 2018; Shi et al., 2019; Alam et al., 2020; Lee et al., 2020; Li and Li, 2020). Among them, host-guest doping materials containing heavy atoms are an effective way to obtain efficient RTP by constructing a relatively rigid environment to restrain the nonradiative decay of the triplet state and promoting the ISC through the heavy-atom effect (Bolton et al., 2011; Kabe and Adachi, 2017; Zhang et al., 2019a; Zhang et al., 2019b; Lei et al., 2020; Liu et al., 2021a; Wang et al., 2021a). For example, Bolton et al. (2011). have developed an efficient RTP system with efficiency and lifetime up to 55% and 8.3 ms, respectively, through diluting the aldehyde chromophore into a host crystal with a similar halogen-bonding motif. Liu et al. (2021b) have developed an RTP emission of organic host-guest doped system with fluorescence and phosphorescence efficiency up to ~67.5% and ~13.2%, respectively, in a solid state using a phenylamine derivative containing halogen atoms as a guest and benzophenone as a host. Halogen-bonding is a crucial way to enhance the heavy-atom effect and promote spin-orbit coupling (SOC) to accelerate phosphorescence with high quantum yields in organic materials (Qu et al., 2019). However, the presence or substitution of heavy halogen atoms seriously affects the chemical stability upon thermal and electrical treatments (Zhou et al., 2018). 

To avoid or overcome these shortcomings, heavy atom free host-guest interactions are an important approach in this research field. For instance, Lei et al. (2019) have developed a series of heavy-atom-free pure organic host-guest doping systems by using 4-(2-(4-(diphenylamino) -phenyl)-2-oxoethyl)benzonitrile with a donor moiety (triphenylamine, TPA) and an acceptor moiety (benzonitrile) within the same molecule as a guest, and TPA and 4-(cyanomethyl) benzonitrile as a donor host and acceptor host respectively, which lead to an enhanced fluorescence (Φ = 63~76%) and RTP (Φ = 7.6~14.5%, τ = 119~317 ms) under ambient conditions. In another instance, Wang et al. (2020) have successfully developed an efficient heat-responsive RTP material with three components by utilizing N,N-dimethylpyridin-4-amine as a host and a blue RTP molecule (N,N,N,N-tetramethylbenzidine as a guest, and energy acceptor (fluorescein) with phosphorescence efficiency and lifetime of up to 13.4% and 2.08 s, respectively, through the simple host-guest doping strategy. Furthermore, Wang et al. (2021b) have also developed a series of efficient pure organic RTP materials by using phenothiazine derivatives (CzS-CH_3_ and CzS-C_2_H_5_) as guests and their corresponding dioxide derivatives (CS-CH_3_ and CS-C_2_H_5_) as hosts, showing high phosphorescence efficiency (43%) as well as a long afterglow of up to 25 min in an aqueous environment. As can be seen from the above analysis, the phosphorescence efficiency of host-guest doping materials without heavy atoms is lower than that of RTP materials containing heavy atoms. Therefore, it remains a challenge to further improve the phosphorescent quantum efficiency of RTP materials without heavy atoms.

In the present study, we have developed a heavy atom free pure organic host-guest doped material, in which thianthrene (TTR) was selected as a guest molecule and benzophenone (BPO) as a host. TTR was selected as the guest because it contains dual-heteroatom of sulfur with abundant lone-pair electrons which can effectively facilitate the SOC and further increase the intersystem crossing (Lei et al., 2019; Zhao et al., 2020). BPO was chosen as the host due to the characteristics of low melting point, good crystallinity, and phosphorescent features. According to the literature (Chen et al., 2020; Liu et al., 2021b), the host matrix is believed to play a synergistic role in energy transfer during the phosphorescence emission of the guest molecule, so the abundant lone-pair electrons of TTR are obviously beneficial for energy transfer. The low melting point of BPO (48.5°C) promotes the preparation of the doped materials by the melt-casting method, and the good crystallinity is beneficial to provide a rigid environment for the guest molecule (Chen et al., 2020). As we expected, a series of host-guest doped crystals all showed excellent room-temperature phosphorescence (RTP). Interestingly, the multi-emission (phosphorescence) peaks of individual guest (BPO) molecules nearly became a single emission peak, especially at a 100:1 molar ratio of the host-guest doped system, which presented an efficient RTP emission with 46% quantum efficiency and a long lifetime of up to 9.17 ms under ambient conditions. This work will provide an effective way to further design new organic doping systems with RTP.

## 2 Materials and Methods

Reagents and materials: Unless otherwise noted, all reagents used in the experiments were purchased from commercial sources without further purification. For flash column chromatography, silica gel with 200–300 mesh was used.

Measurements: The Steady-state luminescence, delayed luminescence spectra, and lifetimes were recorded on an Edinburgh Instruments LTD FLS1000 photoluminescence spectrometer, which is equipped with a xenon arc lamp, xenon flash lamp (μF2), picosecond pulsed diode laser (EPL), and picosecond pulsed led (EPLED). The delayed luminescence spectra were collected with a delay time of 1 or 8 ms. Photoluminescence efficiency was collected on a Hamamatsu Absolute PL Quantum Yield Spectrometer C11347. Luminescent photos were taken by a Canon EOS 700D camera. Powder X-Ray diffraction (XRD) patterns at room temperature were measured on an X-ray diffractometer (RIGAKU, RINT-ULTIMA III) using Cu Kα radiation (λ = 1.54051 Å). All the measurements of the photophysical properties for emissive materials are made carried out in air.

## 3 Results and Discussions

**FIGURE 1:**
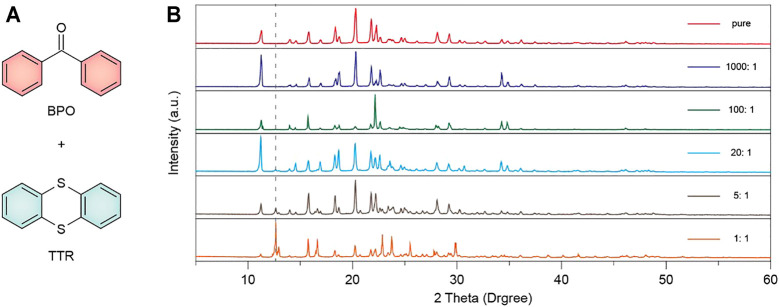
**(A)** Chemical structures of the host matrices BPO and guest emitter TTR. **(B)** Powder XRD patterns of pure BPO and BPO/TTR doped crystalline materials at different molar ratios (1000:1, 100:1, 20:1, 5:1 and 1:1 Mol %).

The molecular structures of TTR (m. p. 151–155°C) and BPO (m. p. 48.5°C) are shown in Figure 1A. Their doped systems were prepared via a melt-casting method (within a temperature range of 453 K to 395 K) by taking different molar ratios of the BPO and TTR in a vacuum. The powder X-ray diffraction (XRD) patterns (Figure 1B) reveal that there are no significant changes in the spectra of BPO from 1000:1 to 5:1 molar ratio of BPO/TTR. However, the doped powder with 1:1 molar ratio has some new peaks but there are still all the major peaks (11.2°, 15.8°, 18.3°, 20.3°, 21.7°, 22.2°, 28.1°) of BPO in the spectra. These results suggested that the host and guest molecules are homogeneously cocrystallized in the host lattice at a low doping ratio, while at a high molar ratio of BPO (1:1), both molecules are crystallized individually.

**FIGURE 2:**
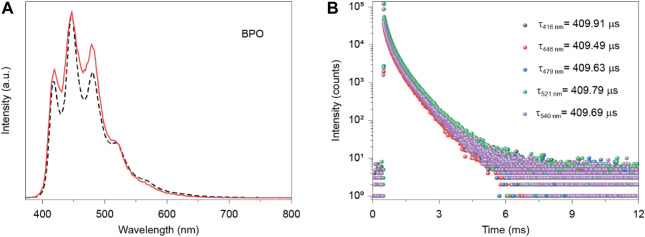
**(A)** Steady-state PL (dotted line) and decay emission spectra (full line) of the host BPO in crystal excited by 383 nm. Inset image show the photograph under 365 nm UV light. **(B)** Lifetime profiles of decay emission bands at 416, 446, 479, 521 and 540 nm for the host (BPO) excited by 396 nm.

**FIGURE 3:**
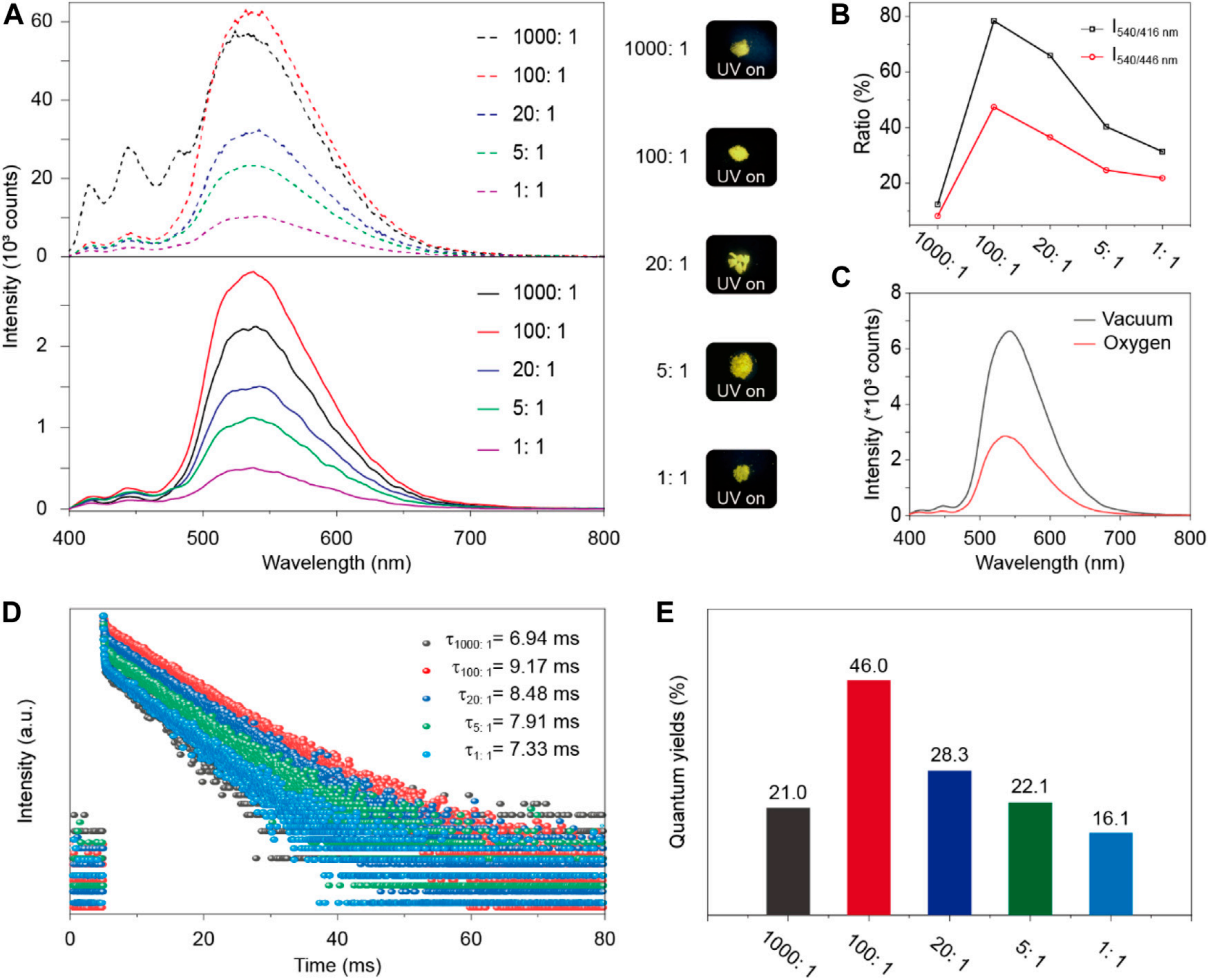
**(A)** Steady-state PL (dotted line) and decay emission spectra (full line) of BPO/TTR-doped crystalline powders at different molar ratios excited by 390 nm. Insets show photographs under 365 nm UV light. **(B)** The ratio of phosphorescent intensity at 540 nm and fluorescent intensity at 416 or 446 nm of the decay emission spectra in the BPO/TTR doping system with different doping ratios. **(C)** Phosphorescence spectra of BPO/TTR composite (100:1) under oxygen and vacuum atmosphere, respectively. **(D)** Lifetime profiles of decay emission band at 540 nm for the BPO/TTR composites excited by 390 nm. **(E)** Phosphorescence quantum yields of BPO/TTR-doped materials with different molar ratios of guest (1000:1, 100:1, 20:1, 5:1 and 1:1 Mol %).

Next, their photophysical properties were studied through steady-state photoluminescence (PL) spectra, and time-resolved emission decay curves in solid state. Pure crystalline BPO powders showed almost identical multiple peaks for steady-state photoluminescence (PL) and phosphorescence with emission peaks at 416 nm (409.91 μs), 446 nm (409.49 μs), 479 nm (409.63 μs) and a shoulder band around 521 nm (409.79 μs) under ambient conditions (Figures 2A,B). After doping with TTR, their blend crystalline powders exhibit a new emission band at 540 nm in both steady-state PL and phosphorescence spectra with a slight red shift of pure BPO (Figure 3A). Moreover, the phosphorescence spectra of blend crystalline powders became almost a single emission band at 540 nm, indicating that the energy transfer between singlet-triplet states from BPO to TTR may occur. It can significantly improve the phosphorescence properties of the doping system. (Lei et al., 2019; Lei et al., 2020) Besides, the doped materials exhibit bright yellow emission under the UV lamp excitation at 365 nm. Moreover, from the ratio of phosphorescent intensity at 540 nm and steady-state PL intensity (416 nm and 446 nm) in the BPO/TTR systems, we can find that different doping concentrations have different influences on the photophysical properties of organic room temperature phosphorescence (Figure 3B). Notably, compared with the phosphorescence in a vacuum, the intensity of the phosphorescence emission band around 540 nm significantly decreased in an oxygen atmosphere, indicating the phosphorescence feature.

It is noteworthy to mention that when guest molecules were doped into the host material with the molar ratio of 1:100, the characteristic steady-state PL and phosphorescence emission peaks (at 416, 446, 479, and 521 nm) of the host material (BPO) were reduced to their lowest level compared to other doped molar ratios (1:1000, 1:20, 1:5 and 1:1). Besides, the phosphorescence lifetime of all doped materials is higher than the individual host (BPO) at their maximum emission peak at 540 nm and the doped material with a 100:1 molar ratio showed the longest emission lifetime of 9.17 ms (Table 1 and Figure 3D). From the performance of 1:100 doped molar ratio, it is not only indicated that the doping system does not disturb the original structure of the host which provides a suitable rigid environment, but also it can form a suitable co-crystallized system that effectively facilitates electron SOC and ISC transitions of molecules, increase the rate of intersystem crossing (S_1_→T_1_) and accelerate the subsequent T_1_→S_0_ phosphorescence processes (Qu et al., 2019).

**TABLE 1:** Photophysical properties of the BPO/TTR-doped materials at different molar ratios of guest.

Samples (host:guest)	*λ*_*em*_ (nm)	*Ф*_phos._ (%)	τ (ms)
BPO	416	-	0.410
	446	-	0.409
	479	-	0.410
	521	-	0.410
1000:1	416	-	0.142
	446	-	0.156
	540	21%	6.94
100:1	416	-	0.233
	446	-	0.230
	540	46%	9.17
20:1	416	-	0.104
	446	-	0.105
	540	28.3%	8.48
5:1	416	-	0.089
	446	-	0.081
	540	22.1%	7.91
1:1	416	-	0.082
	446	-	0.097
	540	16.1%	7.33

Moreover, it was surprising to find out that the characteristic peaks of host materials almost disappeared, and a new band at 540 nm appeared after doping the guest materials. This unusual phenomenon can be initially understood by the El-Sayed rule (El Sayed, 1964; Zhou et al., 2018) that the ISC process takes place more easily due to the distinct transition pattern between singlet and triplet states. The dual-heteroatom of sulfur in TTR and carboxylic oxygen in BPO are playing a similar role as that of heavy atoms, which provide the abundant lone pair electrons to strengthen SOC and to facilitate the ISC process between n→π* and π→π* transition states according to the El-Sayed rule^.^(El Sayed, 1964; Zhou et al., 2018)

The emission wavelength and quantum yield of phosphorescence can be tuned by optimizing the doping ratio of the guest (TTR) molecule. Among these experiments, the doped material with a molar ratio of 100:1 presented the highest phosphorescence quantum yields of up to 46% (Figure 3E), which is consistent with the declining trend of characteristic fluorescence and phosphorescence peaks of BPO, due to the spin-orbit coupling to accelerate both the S_1_→T_1_ and subsequent T_1_→S_0_ phosphorescence processes.

**FIGURE 4:**
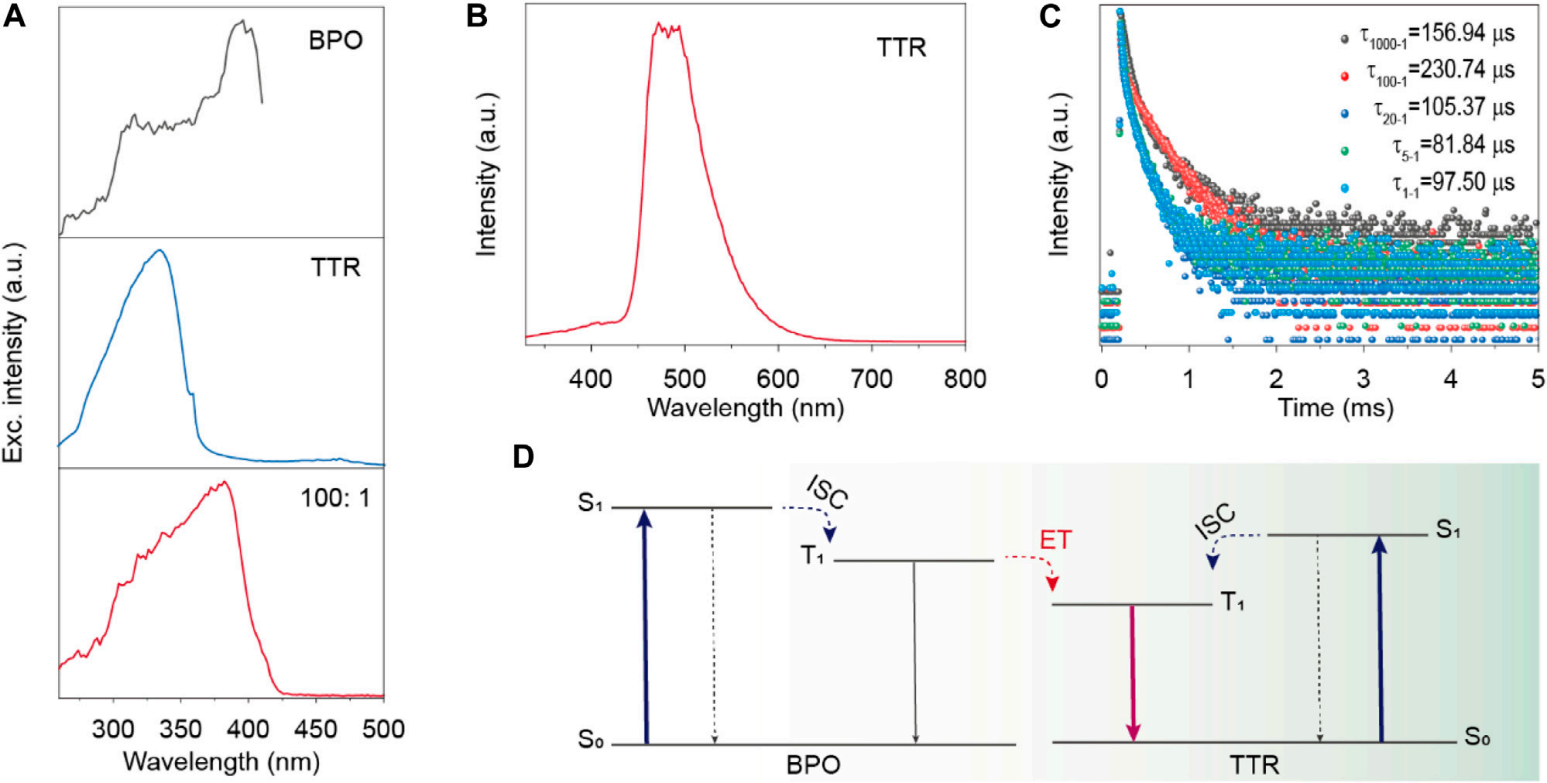
**(A)** Excitation spectra of the BPO, TTR and BPO/TTR composite (1000:1) in solid state under ambient conditions. **(B)** Phosphorescence spectrum of the TTR molecules in dilute *m*-THF solution at 77 K. **(C)** Lifetime profiles of decay emission band at 446 nm for the BPO/TTR composite excited by 390 nm. **(D)** A plausible mechanism for the RTP in BPO/TTR composite.

To gain insight into the mechanism of the highly efficient RTP for BPO/TTR composite, we conducted a series of control experiments including excitation and phosphorescence spectra of BPO, TTR, and composites, as well as luminescent lifetime change. From Figure 4A, it is easily found that the excitation spectra of the BPO and BPO/TTR doped systems were similar, which is different from that of TTR in solid state, indicating the phosphorescence emission band around 540 nm in BPO/TTR composite was related to the BPO host material. Taking the different lifetimes of the emission bands at 540 nm for BPO host and BPO/TTR composite (Figures 2B, 3D) and the phosphorescence spectrum for TTR in an isolated state (Figure 4B) together, we concluded that the phosphorescence emission around 540 nm stemmed from the isolated TTR molecules in BPO/TTR composite. It is worth noting that the lifetimes of emission bands at 446 nm gradually decrease with the ratio of TTR guests increasing (Figure 4C), suggesting that there existed energy transfer from BPO host to TTR guest molecules. To sum up, the RTP was ascribed to the phosphorescence emission of the isolated TTR molecules in crystalline BPO/TTR composite (Figure 4D). Both crystalline molecular environment and efficient energy transfer between host and guest molecules significantly boost the phosphorescence for high efficiency.

## 4 Conclusion

In conclusion, we have developed an efficient RTP doping material based on two heavy atom free organic small molecules, benzophenone and thianthrene. The doped materials have contained different heteroatoms (TTF contains dual S atoms and BPO contains O atom) with abundant lone pair electrons, which make them promising candidates to enhance SOC, leading to an efficient ISC in pure organic metal-free RTP materials, instead of commonly used heavy atoms. We achieved an efficient RTP emission with phosphorescence efficiency of up to 46% under ambient conditions by tuning the doping molar ratio of the host and guest molecules. Our work not only provides a new approach to develop heavy atom free pure organic host-guest doped RTP materials but also expands the methods to enhance their quantum efficiency.

## Data Availability

The original contributions presented in the study are included in the article/supplementary material, further inquiries can be directed to the corresponding author.
